# Different biomechanical effects of clear aligners in bimaxillary space closure under two strong anchorages: finite element analysis

**DOI:** 10.1186/s40510-022-00435-2

**Published:** 2022-11-14

**Authors:** Jun-qi Liu, Guan-yin Zhu, Yi-gan Wang, Bo Zhang, Shuang-cheng Wang, Ke Yao, Zhi-he Zhao

**Affiliations:** 1 State Key Laboratory of Oral Diseases and National Clinical Research Center for Oral Diseases, West China Hospital of Stomatology, Sichuan University, No. 14, 3rd section, Ren Min Nan Road Chengdu, Chengdu, 610041 Sichuan China; 2 Department of Orthodontics, West China Hospital of Stomatology, Sichuan University, Chengdu, China

**Keywords:** Clear aligners, Finite element analysis, Extraction space closure, Strong anchorage, Biomechanics

## Abstract

**Background:**

Clear aligner (CA) treatment has been gaining popularity, but the biomechanical effects of CAs in bimaxillary dentition have not been thoroughly investigated. Direct and indirect strong anchorages are two common anchorage control methods, but the underlying biomechanical mechanism has not yet been elucidated. This study aimed to investigate the different biomechanical effects of CAs in closing the bimaxillary space under different anchorage controls, further instructing the compensation strategies design and strong anchorage choice in clinical practice.

**Methods:**

Three-dimensional (3D) bimaxillary models of different anchorage controls were created based on cone-beam computed tomography and intraoral scan data. Four first premolars were extracted using 3D modeling software. Finite element analysis was conducted to simulate the space closure process of the CAs.

**Results:**

In the two strong anchorage groups, the bimaxillary dentition presented different movement patterns during the space closure process, and the lower dentition was more vulnerable to elastic force. From the vertical view, direct strong anchorage with elastic force had the advantage of flattening the longitudinal occlusal curve and resisting the roller-coaster effects, whereas indirect strong anchorage could lead to a deep longitudinal occlusal curve. From the sagittal view, indirect strong anchorage with metallic ligaments had a greater instantaneous anchorage protection effect, particularly in the lower dentition, which reduced the mesial movement of the posterior teeth by nearly four times that of the direct anchorage group. In addition, indirect strong anchorage presented better anterior teeth torque/tipping control, while direct strong anchorage could aggravate lingual tipping of the upper central incisors. Due to the differences in anterior–posterior anchorage and arch shape, compared with the upper dentition, anchorage preservation and vertical control effects were amplified in the lower dentition.

**Conclusions:**

The biomechanical effects of CAs differed between the two strong anchorage groups. Due to the differences in dentition morphology, anterior–posterior anchorage, and dental arch shape, CAs present different biomechanical effects in bimaxillary space closure. Orthodontists should consider the corresponding mechanical compensation according to specific anchorage control methods and dentitions.

**Supplementary Information:**

The online version contains supplementary material available at 10.1186/s40510-022-00435-2.

## Background

Orthodontists and patients have increasingly favored clear aligner (CA) treatment in recent years due to its advantages in terms of comfort, esthetics, and better oral hygiene [1, 2]. In addition, compared with traditional fixed appliances, CA treatment is superior in molar distalization, space closure, and anterior tooth alignment and presents satisfactory treatment effects [3, 4]. However, CA treatment underperforms in deep overbite correction, arch expansion, and root movement control, making it less suitable for complex cases involving massive tooth movement, such as extraction cases [5, 6].

Indeed, compared to fixed appliances, CAs have a completely different biomechanical system. The orthodontic force of CAs is generated by the resilience of the viscoelastic polymer materials. By wearing CAs, the teeth are expected to be moved to a series of predesigned positions. However, this target cannot be easily achieved. First, the homogeneous polymer material cannot exhibit sufficient strength and elasticity to ensure full movement of the teeth to the designated position. In addition, viscoelastic materials undergo creep and stress relaxation with prolonged wear, resulting in the continuous attenuation of orthodontic forces [7, 8]. In addition to the mechanical properties, good retention performance is also important for treatment efficacy. The retention performance of CAs mainly relies on dentition morphology and varies among different teeth and movement patterns. This factor causes CAs to present specific orthodontic efficacies among different movement patterns [9, 10].

Nowadays, to manage malocclusion and facial esthetics, the proportion of extraction cases has reached 30% [11]. To allow these patients to benefit from CA treatment, multiple strategies have been adopted to compensate for the mechanical and retention deficiencies, for instance, adding extra overcorrection to the target position, designing specific attachments to enhance retentive performance, and using a long power arm to retract the anterior teeth [9, 12–14]. Notably, all these orthodontic strategies should be based on established anchorage control plans. Therefore, investigating the biomechanical characteristics of different anchorage controls would lay the foundation for exploring more strategies for extraction cases.

Anchorage control is not only affected by the control form but also by the dentition morphology of the bimaxillary. Miniscrews are widely used as temporary anchorage devices (TAD). In CA treatment, there are two common TAD-assisted anchorage control methods: direct strong anchorage (the anterior teeth are retracted by elastics) and indirect strong anchorage (the second premolars are fixed by metallic ligation). Our previous studies analyzed the different biomechanical effects of CAs in closing the maxillary extraction space between different anchorage types and provided insights into compensation strategies. At the same time, we found that the tooth morphology, anterior–posterior anchorage difference, and dental arch shape could be important factors that cause distinctive bimaxillary anchorage differences. To date, no study has comprehensively illustrated the different biomechanical effects of CAs on closing the bimaxillary space under different anchorages. Finite element analysis (FEA) is a rational and widely used method to solve this problem. Unlike clinical observation studies, which could be influenced by confounding factors, FEA, which is based on a stable and reproducible medical model, has become a reliable research method for investigating orthodontic biomechanics [15, 16].

In this study, we conducted FEA on a standard first premolar extraction bimaxillary model under moderate anchorage, direct strong anchorage, and indirect strong anchorage controls. Based on the FEA results, we compared the biomechanical effects of CAs on bimaxillary space closure under different anchorage controls, especially between moderate anchorage and two strong anchorages. To make the results more straightforward, tooth displacement was extracted and converted into a tooth movement table, which is in line with the current CA design systems, such as ClinCheck of Invisalign (Align Technology, Santa Clara, CA, USA). In addition, the center of rotation of each tooth was analyzed to illustrate the tooth movement pattern. Finally, potential strategies for compensation design and strong anchorage choice are discussed.

## Material and methods

Cone-beam computed tomography (CBCT) and intraoral scan data were obtained from the orthodontic case archive of the West China Hospital of Stomatology, Sichuan University. The inclusion criteria were as follows: (1) permanent dentition; (2) normal tooth morphology; (3) no obvious crowding; (4) normal tooth torque and occlusal curve; and (5) complete CBCT and intraoral scan data. The exclusion criteria were periodontal and systemic diseases that affected bone development.

**Fig. 1:**
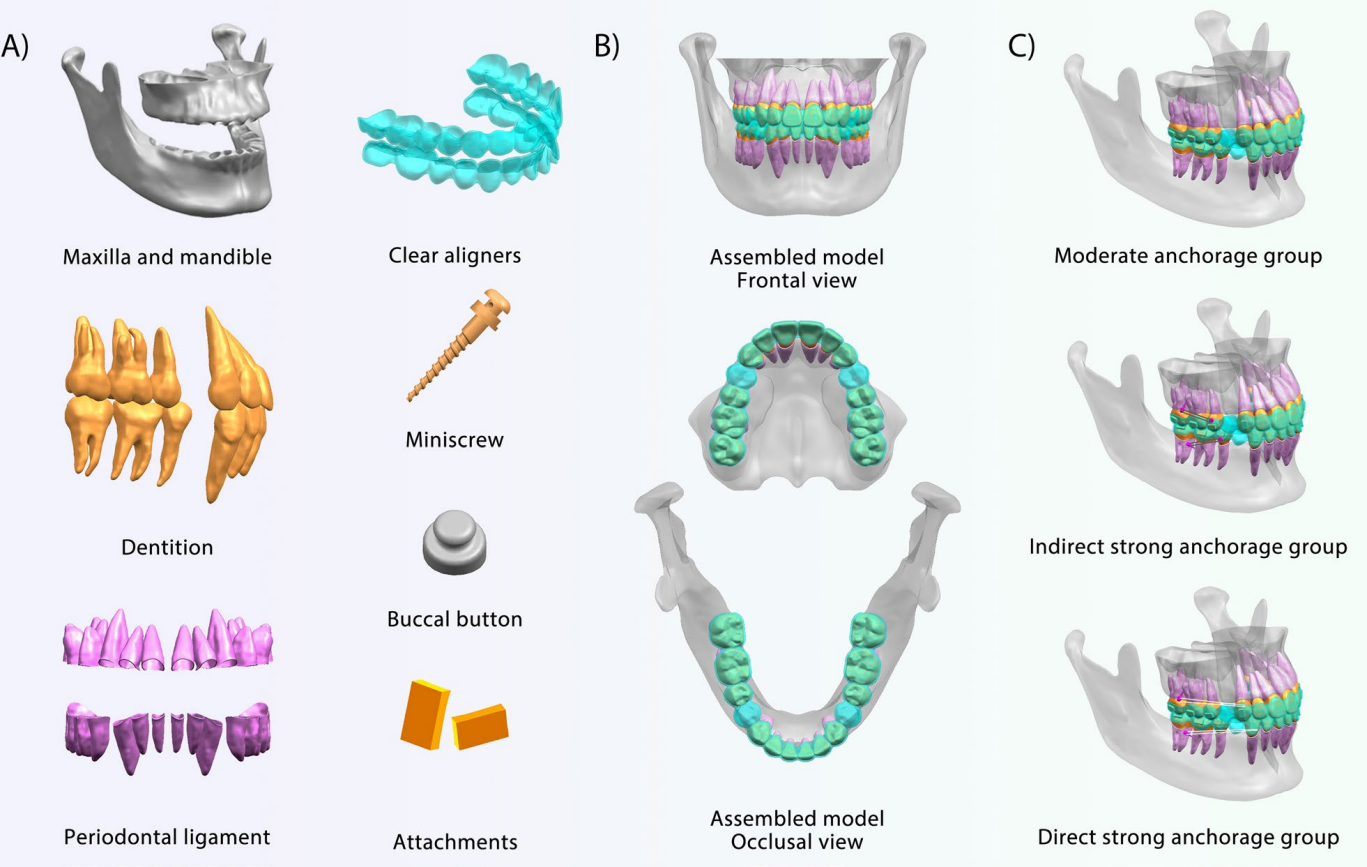
Models for the finite element analysis. **A** The bimaxillary tissues were generated according to patient data, and the clear aligners, attachments, miniscrew, and buccal button were constructed based on available products. **B** The assembled research model in different views. **C** Three groups of different anchorage were constructed: the moderate anchorage group was used as a control group to the two strong anchorage groups. Indirect strong anchorage group: the second premolars and miniscrew were ligated together with metallic ligation wire. Direct strong anchorage group: the elastic force was applied to the canine region of the CA with the assistance of miniscrews

CBCT data (Digital Imaging and Communications in Medicine format) were imported into Mimics Research (version 19.0; Materialise NV, Leuven, Belgium), in which the soft tissues were removed, and the bimaxillary bone and dentition were isolated via threshold segmentation. The obtained 3D geometric models were imported into Geomagic Wrap 2017 (Raindrop Geomagic, 3D Systems, NC, USA) for refinement and smoothing. Intraoral scan data were used to refine the bimaxillary dentition, further improving its precision. A standard bimaxillary model was constructed. The intraoral scan data were exported as STereoLithography files and loaded into Ortho Analyzer (3Shape, Denmark), where the attachments were added, and the clinical crowns were aligned based on Andrews normal occlusion. Then, the CBCT roots were matched to the aligned clinical crowns, and a high-quality dentition model was constructed. Furthermore, four first premolars were removed, and periodontal ligaments were constructed as an even enclosure around the entire tooth root with an average thickness of 0.25 mm [17]. The CAs and attachments were generated in Appliance Designer (3Shape, Denmark), and the CAs were 0.5 mm externally offset from the dental crowns [18]. The miniscrew and buccal buttons were developed using SolidWorks (Dassault Systemes, USA). The miniscrew had a total length of 10.7 mm, 8 mm of which was implanted, consistent with the VectorTAS orthodontic implant (Ormco, USA) (Fig. 1A). Finally, the constructed components were assembled in Unigraphics NX (UG, Siemens PLM Software) as research models (Fig. 1B).

Three anchorage groups were established: (1) moderate anchorage; (2) direct strong anchorage: elastic force was applied to the canine region of CAs through the miniscrews; and (3) indirect strong anchorage: the second premolars and miniscrews were ligated with metallic ligation wire. In detail, in the moderate anchorage, the extraction spaces were closed by aligner contraction. In the strong anchorage groups, anchorages were enhanced by miniscrews which were implanted between the first molars and the second molars, 3–5 mm from the alveolar crest, and at an angle of 30–45° to the long axis of the tooth. The direct strong anchorage referred to the application of elastics on the cutout of the CAs in the canine mesial cervical region. The indirect strong anchorage was a common passive anchorage form that was achieved by ligating the button on the second premolar and the miniscrew with metallic ligation wire. The buttons were bonded close to the buccal gingival line of the second premolars. The attachment design was consistent with the clinical practice. Given the mechanical compensation needs and the dentition morphologies, vertical rectangular attachments were added to the canines and horizontal rectangular attachments were added to the posterior teeth to enhance the root control in the sagittal plane and prevent vertical CA detachment in the middle of the dental arch. The assembled groups are depicted in Fig. 1C. To better illustrate the effect of the elastic force in the direct strong anchorage group, we further removed the CA contraction force and created pure 150 g and 500 g elastic force groups.

The assembled models were imported into ABAQUS 6.14 (Dassault Systemes, Providence, Rhode Island, USA) to develop a 3D finite element model through a meshing and discretization process. The linear mesh was converted into tetrahedral elements (C3D10), and the discretization parameters are listed in Table 1. The model components were set as isotropic, homogeneous, and linearly elastic, and the material properties were obtained from previous studies [14, 19–21] (Table 2). To prevent the model from bodily motion when the force was loaded, the bases of the maxilla and mandibular condyle were fixed. A frictional coefficient contact condition (μ) of 0.2 was set between the CAs and dentitions [21]. Space closure was simulated using the thermal contraction method. In detail, 1 mm of the extraction space was selected, and 0.2 mm of the selected area was fully contracted by changing the temperature difference and linear expansion coefficient parameters (Additional file 1: Fig. S1). After setting the loading configuration, the FEAs were conducted using ABAQUS 6.14, and the nodal displacements and von Mises values were obtained.

**Table 1:** Discretization parameters

Group	Total no. of nodes	Total no. of elements
Moderate anchorage group (maxilla)	860,135	478,629
Moderate anchorage group (mandible)	943,028	537,453
Direct anchorage group (maxilla)	887,956	495,736
Direct anchorage group (maxilla)	952,692	542,849
Indirect anchorage group (maxilla)	889,572	496,676
Indirect anchorage group (mandible)	951,183	541,938

**Table 2:** Material properties

Material	Young’s modulus, E (Mpa)	Poisson ratio, v
Alveolar bone	1.37*10^4^	0.30
Teeth	1.96*10^4^	0.30
Periodontal ligament	0.69	0.45
Clear aligners	528	0.36
Attachments	1.25*10^4^	0.36
Miniscrew	1.03*10^5^	0.33
Buccal button	2*10^5^	0.30

**Fig. 2:**
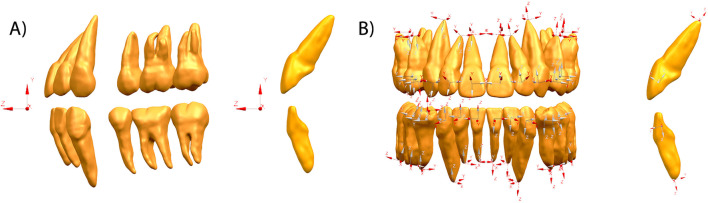
Two coordinate systems were used to evaluate the tooth displacements. **A** Global coordinate system was set based on the occlusal plane and used to evaluate the vertical displacements of teeth. **B** Local coordinate system was set on the body center of the clinical crown and root apex of every tooth: x (mesiodistal direction), y (labial lingual direction), and z (tooth long-axis direction). Local coordinates were used to analyze the labial/lingual and mesial/distal displacements of teeth

In this study, two coordinate systems were used: (1) the global coordinate system, referenced to the occlusal plane, was used to assess the vertical displacement of the teeth; (2) the local coordinate system, set on every tooth, was used to evaluate individual tooth movements in the labial/lingual and mesial/distal directions. Every tooth had two local coordinates, which were located at the center of the tooth crown and root apex point (or center of root apex points for multirooted teeth). The X- and Y-axes represent the labial/lingual and mesial/distal directions, respectively (Fig. 2). Therefore, tooth displacements in the 3D direction can be calculated and extracted as a “tooth movement table,” which is coordinated with the mainstream CA design system. In addition to the 3D tooth movement value, several extra indicators were introduced and calculated: (1) the tipping angle in the buccal/lingual and mesial/distal directions, calculated based on the crown-root length and displacement value of the tooth crown and root; (2) the crown-root movement ratio, the value of the crown movement value divided by the root movement value, was used to reflect the relative positions of the rotation center and root control effect of CAs; the greater the ratio, the better the root control effect; (3) relative incisor extrusion: the displacement differences between the central incisors and posterior teeth (the second premolar and the first molar) in the vertical direction were used to evaluate the vertical control of CAs (Additional file 1: Fig. S2).

## Results

**Fig. 3:**
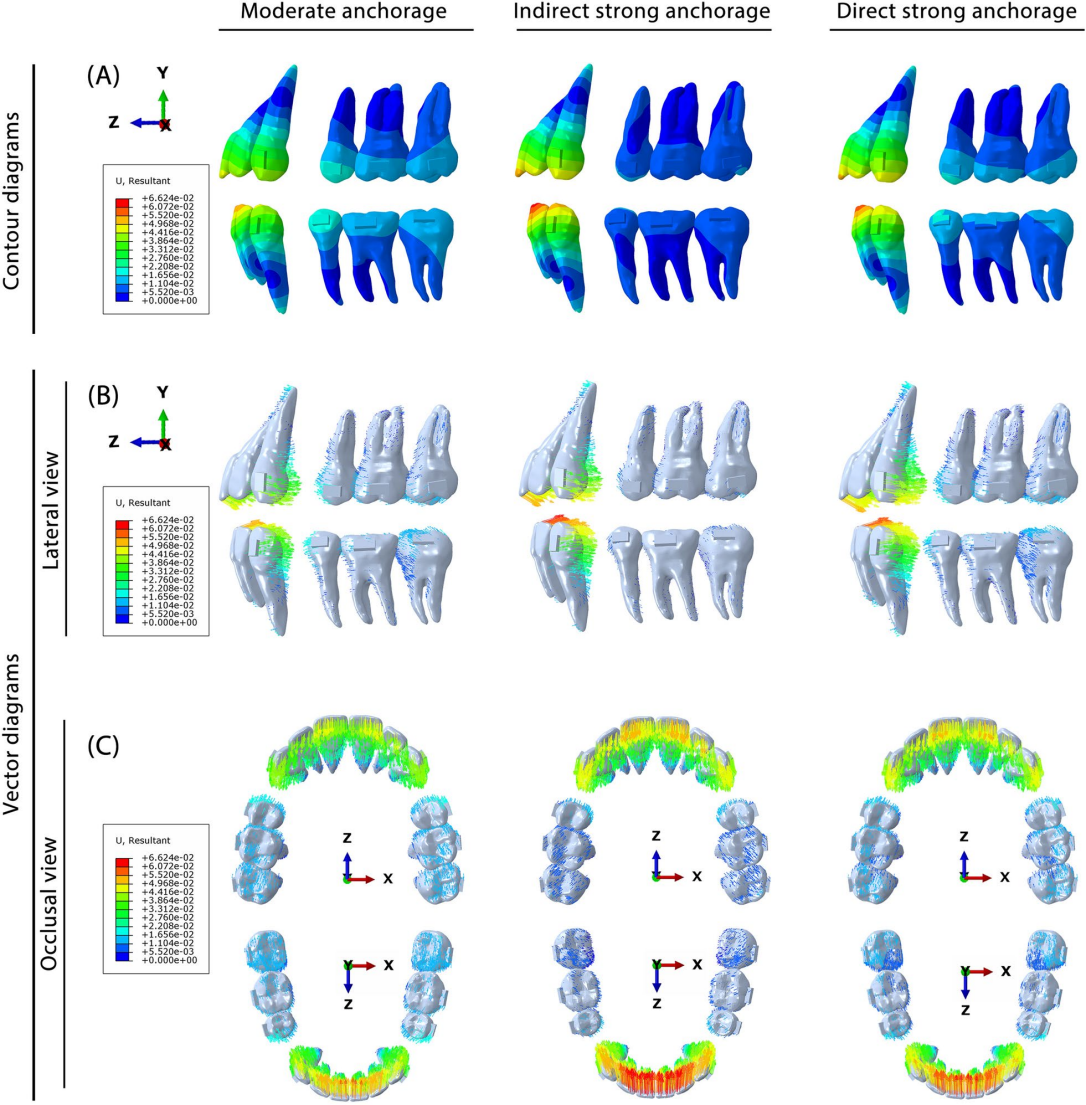
Biomechanical effects of CAs on bimaxillary space closure under different anchorages. **A**) Contour diagram reflecting the displacement tendencies of bimaxillary teeth. **B**, **C** Vector diagram reflecting the displacement directions and the amount of the bimaxillary teeth

The FEA results of the three different anchorage groups are presented in Fig. 3. Both the contour and vector diagrams showed that among the three groups, both the anterior and posterior teeth presented similar movement tendencies but different movement amounts.

**Fig. 4:**
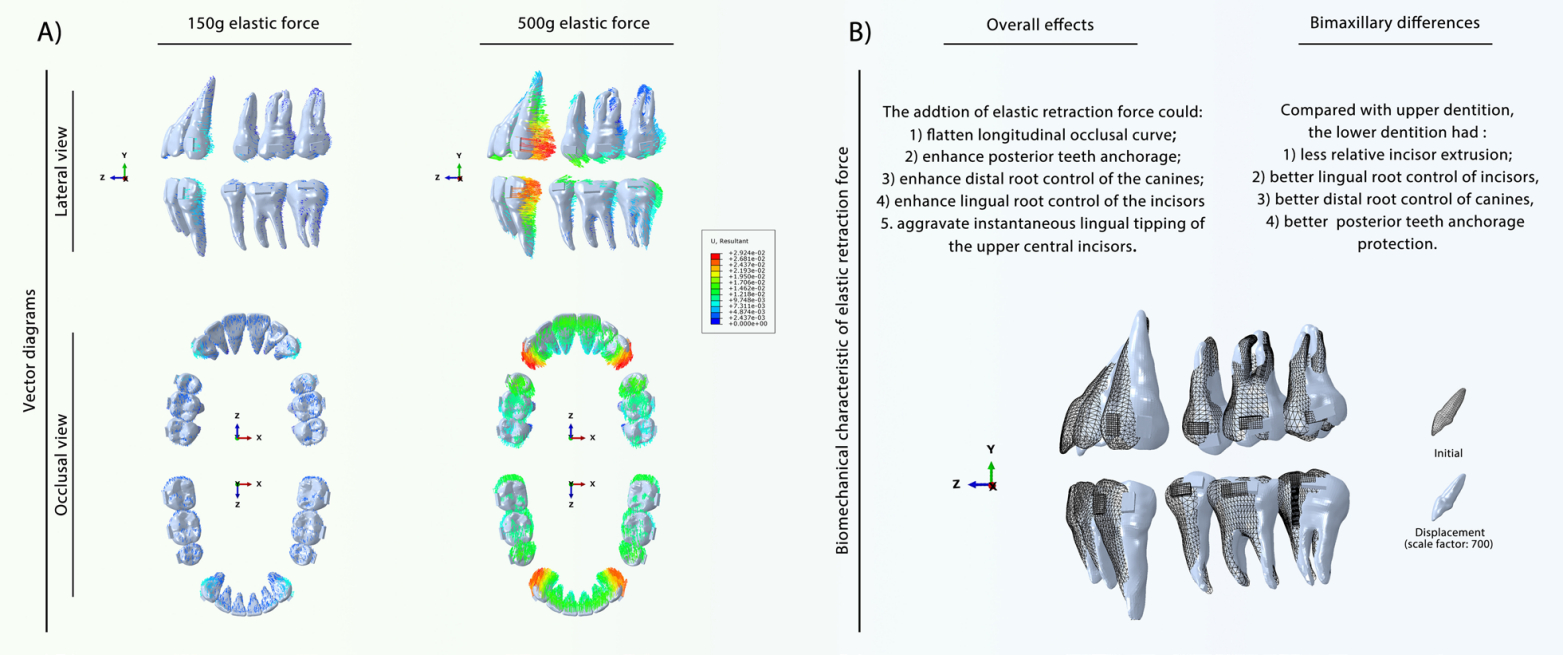
Biomechanical effects of mere elastic force on the bimaxillary space closure. **A** Vector diagram reflecting the displacement directions and distances of teeth displacement under 150 g and 500 g elastic forces. **B** Schematic diagram of different biomechanical effects of elastic force on the upper and lower dentition. The tipping movement and the root control are different concept in this work. The use of elastic force would cause larger instantaneous teeth tipping; the root control was actually enhanced, which means, compared with the moderate group, retracting same distance in direct strong anchorage group could cause less anterior teeth tipping

The vertical and sagittal directions are the two main directions of tooth movement. The vertical tooth movement data are listed in Table 3. This showed that CAs could intrude the second premolars and first molars and extrude the other teeth, deepening the longitudinal occlusal curve of both the upper and lower dentitions. To better analyze the change in overbite in the central incisors, the relative incisor extrusion was calculated (Table 4). The mandibular incisors had more relative incisor extrusion in the three anchorage groups than the maxillary incisors. Compared with the moderate anchorage group, the indirect strong anchorage group showed more relative incisor extrusion, causing a deep overbite tendency, while the direct strong anchorage relieved incisor extrusion, which was attributed to the vertical component of the elastic force. To further confirm the biomechanical effects of elastic force, pure 150 g and 500 g elastic forces without CA contraction were used (Fig. 4A). 150 g is a commonly used orthodontic force in clinic practice, while 500 g belongs to heavy force close to the maximum force used in the clinic. Therefore, 150–500 g elastic forces should have covered the orthodontic force range. The investigation of these two elastic forces was aimed at confirming if the tooth movement pattern changed depending on the strength of the force. The results showed that the 150 g and 500 g elastic forces had similar biomechanical effects; within the range of orthodontic force, the elastic force only changed the instantaneous displacement of the tooth and did not change the tooth movement pattern. This result indicated that the effect of the elastic force was stable and regular: the larger the force, the greater the effect. According to the vertical tooth displacement data (Table 5, Additional file 1: Table S1), the elastic force can extrude the middle dental arch (the second premolar and the first molar) and intrude the two ends of the dental arch (the anterior teeth and the second molars), which is the desired effect for resisting the roller-coaster effect. In addition, when applying a similar force, the lower dentition showed a more obvious vertical control effect in the anterior teeth than the upper dentition. These biomechanical effects are summarized in Fig. 4B.

**Table 3:** Vertical movement of teeth in different groups

	Anterior teeth			Posterior teeth		
	1	2	3	5	6	7
Moderate anchorage group (maxilla)	1.381E	0.981E	0.339E	− 0.318I	− 0.167I	0.686E
Moderate anchorage group (mandible)	1.720E	1.264E	0.344E	− 0.342I	− 0.079I	0.827E
Indirect anchorage group (maxilla)	1.562E	1.115E	0.404E	− 0.512I	− 0.134I	0.429E
Indirect anchorage group (mandible)	1.738E	1.255E	0.323E	− 0.652I	− 0.143I	0.295E
Direct anchorage group (maxilla)	1.407E	0.849E	0.088E	− 0.272I	− 0.143I	0.581E
Direct anchorage group (mandible)	1.635E	1.086E	0.144E	− 0.275I	− 0.068I	0.684E

+ values indicate extrusion of teeth (× 10^−2^ mm); − values indicate intrusion of teeth (× 10^−2^ mm); E, tooth extrusion based on the occlusal plane; I, tooth intrusion based on the occlusal plane; 1, central incisor; 2, lateral incisor; 3, canine; 5, second premolar; 6, first molar; 7, second molar

**Table 4:** Relative incisor extrusion in different groups

Group	Relative to the second premolars (× 10^−2^ mm)	Relative to the first molars (× 10^−2^ mm)
Moderate anchorage group (maxilla)	1.699E	1.548E
Moderate anchorage group (mandible)	2.062E	1.800E
Indirect anchorage group (maxilla)	2.074E	1.696E
Indirect anchorage group (mandible)	2.390E	1.882E
Direct anchorage group (maxilla)	1.679E	1.550E
Direct anchorage group (mandible)	1.910E	1.703E

The values indicate the relative incisor extrusion of teeth (× 10^−2^ mm); E, tooth extrusion based on the occlusal plane; the relative incisor extrusion is calculated based on the displacement differences between the central incisors and posterior teeth (the second premolar and the first molar) in the vertical direction

**Table 5:** Vertical movement of teeth in the 150 g elastic force group

Tooth	Anterior teeth				Posterior teeth		
	1	2	3		5	6	7
Group				Maxilla			
Extrusion/Intrusion (× 10^−2^ mm)	− 0.009I	− 0.081I	− 0.045I		0.160E	0.050E	− 0.068I
Relative incisor extrusion to the second premolars				− 0.169I			
Relative incisor extrusion to the first molars				− 0.059I			

Tooth	Anterior teeth				Posterior teeth		
	1	2	3		5	6	7
Group				Mandible			
Extrusion/Intrusion (× 10^−2^ mm)	− 0.109I	− 0.141I	− 0.063I		0.162E	0.034E	− 0.124I
Relative incisor extrusion to the second premolars				− 0.272I			
Relative incisor extrusion to the first molars				− 0.144I			

+ values indicate extrusion of teeth (× 10^−2^ mm); − values indicate intrusion of teeth (× 10^−2^ mm); E, tooth extrusion based on the occlusal plane; I, tooth intrusion based on the occlusal plane; 1, central incisor; 2, lateral incisor; 3, canine; 5, second premolar; 6, first molar; 7, second molar; the relative incisor extrusion is calculated based on the displacement differences between the central incisors and posterior teeth (the second premolar and the first molar) in the vertical direction

Sagittal movement is another important form of tooth movement that occurs during extraction space closure. As shown in Fig. 3B–C, compared with the moderate anchorage group, the two strong anchorage groups had less mesial movement of the posterior teeth and more displacement of the anterior teeth, demonstrating that both strong anchorage forms were effective in posterior teeth anchorage protection. The detailed mesial displacements of the posterior teeth are presented in Table 6. It was found that the upper posterior teeth generally presented greater anchorage loss than the lower posterior teeth in all three groups. Compared with the direct strong anchorage group, the indirect strong anchorage group presented greater posterior teeth protection effects. Notably, indirect strong anchorage could apparently enhance the lower posterior teeth anchorage, and it reduced the mesial movement of the posterior teeth by nearly four times that of direct strong anchorage.

**Table 6:** Mesial/distal movement value of the posterior tooth crown

Group	5	6	7
Moderate anchorage group (maxilla)	− 1.402 M	− 1.096 M	− 0.944 M
Moderate anchorage group (mandible)	− 1.227 M	− 1.058 M	− 0.853 M
Indirect anchorage group (maxilla)	− 0.882 M	− 0.668 M	− 0.554 M
Indirect anchorage group (mandible)	− 0.485 M	− 0.299 M	− 0.210 M
Direct anchorage group (maxilla)	− 1.199 M	− 0.943 M	− 0.814 M
Direct anchorage group (mandible)	− 1.020 M	− 0.883 M	− 0.716 M
150 g elastic force group (maxilla)	0.254D	0.225D	0.202D
150 g elastic force group (mandible)	0.312D	0.280D	0.245D

+ values indicate distal movement of the tooth crown (× 10^−2^ mm); − values indicate mesial movement of the tooth crown (× 10^−2^ mm); M, mesial movement; D, distal movement; 5, second premolar; 6, first molar; 7, second molar

**Fig. 5:**
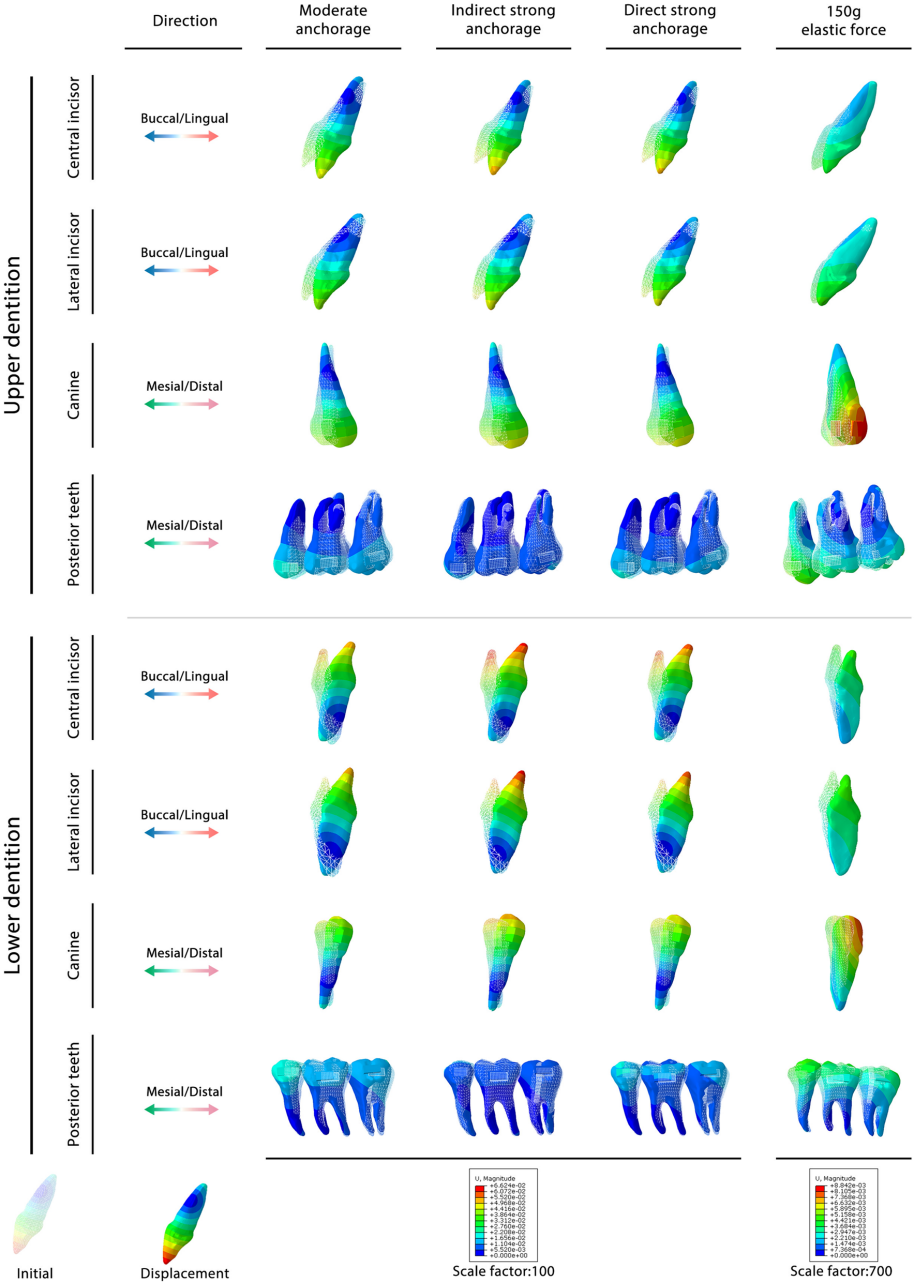
Tipping movement tendency of the bimaxillary teeth under different anchorage control and elastic force. 1, central incisors; 2, lateral incisors; 3, canines; 5, second premolar; 6, first molar; 7, second molar

Due to the control deficiencies of CAs, tipping movement is the most common movement pattern. As shown in Fig. 3, the tipping movement commonly occurred in the sagittal direction. These tipping movements mainly manifested in lingual tipping of the incisors, distal tipping of the canines, and mesial tipping of the posterior teeth (Fig. 5; Table 7). The bimaxillary dentition had the same tipping movement tendency but showed different tipping amounts. Generally, the lower anterior teeth showed the most obvious tipping movements, including lingual tipping of the incisors and distal tipping of the canines. The lower posterior teeth presented less mesial tipping movement than the upper dentition, which could be attributed to the greater root anchorage difference. In contrast to the moderate anchorage group, the two strong anchorage groups had more tipping movements of the anterior teeth and less mesial tipping movements of the posterior teeth. Compared with the direct anchorage group, the indirect strong anchorage group showed more tipping movement of the anterior teeth and second premolars but less mesial inclination of the molars (Table 7). In the direct anchorage group, elastic force promoted lingual inclination of the incisors and distal tipping movement of the canines and posterior teeth.

**Table 7:** Significant tipping movement of teeth in different anchorage groups

Tooth	Anterior teeth			Posterior teeth		
	1	2	3	5	6	7
Moderate anchorage group (maxilla)	− 14.440L	− 13.186L	12.962D	− 7.356 M	− 5.895 M	− 4.956 M
Moderate anchorage group (mandible)	− 23.441L	− 18.700L	13.800D	− 5.423 M	− 4.190 M	− 3.725 M
Indirect anchorage group (maxilla)	− 16.201L	− 14.733L	14.452D	− 6.728 M	− 3.818 M	− 3.127 M
Indirect anchorage group (mandible)	− 25.876L	− 20.421L	15.198D	− 4.986 M	− 2.172 M	− 1.755 M
Direct anchorage group (maxilla)	− 15.828L	− 13.789L	13.951D	− 6.326 M	− 4.995 M	− 4.207 M
Direct anchorage group (mandible)	− 25.605L	− 20.155L	14.913D	− 4.551 M	− 3.482 M	− 3.053 M
150 g elastic force group (maxilla)	− 1.308L	− 0.842L	1.294D	1.602D	1.349D	1.220D
150 g elastic force group (mandible)	− 1.476L	− 1.236L	1.109D	1.491D	1.202D	1.220D

+ Values indicate the tipping movement in the buccal or distal direction (× 10^−2^ mm); − values indicate the lingual or mesial direction (× 10^−2^ mm); the direction is also marked after the value. B, buccal movement; L, lingual movement; M, mesial movement; D, distal movement. 1, central incisor; 2, lateral incisor; 3, canine; 5, second premolar; 6, first molar; 7, second molar. The calculation of buccal/lingual and mesial/distal tipping change is described in the Methodology section

To further investigate the tipping movement pattern and root control effect of the CA, this study analyzed the displacement of the rotation center of each tooth. As shown in Fig. 5, the rotation centers were commonly located between the tooth crown and root. Theoretically, the closer the rotation center is to the tooth root, the better the root control effect (the center of rotation is further away from the center of resistance), and less tipping occurs when the teeth displace the same distance. Therefore, according to the crown-root movement ratio, the use of indirect strong anchorage could relieve lingual tipping of the incisors and distal tipping of the canines compared with moderate anchorage, which was more obvious in the lower dentition (Table 8). The use of direct strong anchorage could effectively relieve distal tipping of the canines and lingual tipping of the incisors, except for the upper central incisors. For posterior teeth, the use of indirect strong anchorage could aggravate mesial tipping of the posterior teeth, particularly in the lower dentition. In contrast, direct anchorage showed an advantage in relieving the mesial tipping of the posterior teeth, and this effect could be amplified in the lower dentition.

**Table 8:** Root control effect along the significant tipping direction

Tooth	Anterior teeth			Posterior teeth		
	1	2	3	5	6	7
Moderate anchorage group (maxilla)	2.106L	2.164L	1.818D	2.533 M	2.673 M	3.064 M
Moderate anchorage group (mandible)	2.414L	2.299L	1.653D	3.456 M	7.760 M	8.730 M
Indirect anchorage group (maxilla)	2.147L	2.225L	1.870D	0.974 M	2.175 M	2.356 M
Indirect anchorage group (mandible)	3.149L	3.071L	2.108D	0.500 M	0.936 M	0.879 M
Direct anchorage group (maxilla)	2.093L	2.212L	1.903D	2.482 M	2.826 M	3.280 M
Direct anchorage group (mandible)	2.471L	2.394L	1.790D	3.316 M	8.052 M	11.238 M
150 g elastic force group (maxilla)	2.057L	3.879L	3.200D	1.477D	1.880D	1.906D
150 g elastic force group (mandible)	7.174L	16.461L	11.018D	2.537D	4.484D	3.684D

The root control effect is reflected by the crown-root movement ratio; the calculation is described in the Methodology section. The value with the same direction can be directly compared; the larger the value, the greater the root control ability of the corresponding direction. L, lingual root control ability; M, mesial root control ability; D, distal root control ability. 1, central incisor; 2, lateral incisor; 3, canine; 5, second premolar; 6, first molar; 7, second molar

## Discussion

The orthodontic efficacy of CA treatment is attributed to its good elasticity and retention. Unlike traditional fixed appliances, which have stable bracket retention and multiple choices of NiTi wires with different mechanical properties, the orthodontic force of CAs is derived from the deformation of the viscoelastic polymer, and retention is derived from its chimeric retention with the tooth. Therefore, a reasonable design of attachments is the basis for CAs to exert desirable orthodontic force [10]. The attachment design in this study has been routinely adopted in clinical practice and was confirmed as effective in root control movement in our previous study. On this basis, we further investigated the influence of anchorage control on bimaxillary dentition, which is closer to what occurs in clinical practice.

**Fig. 6:**
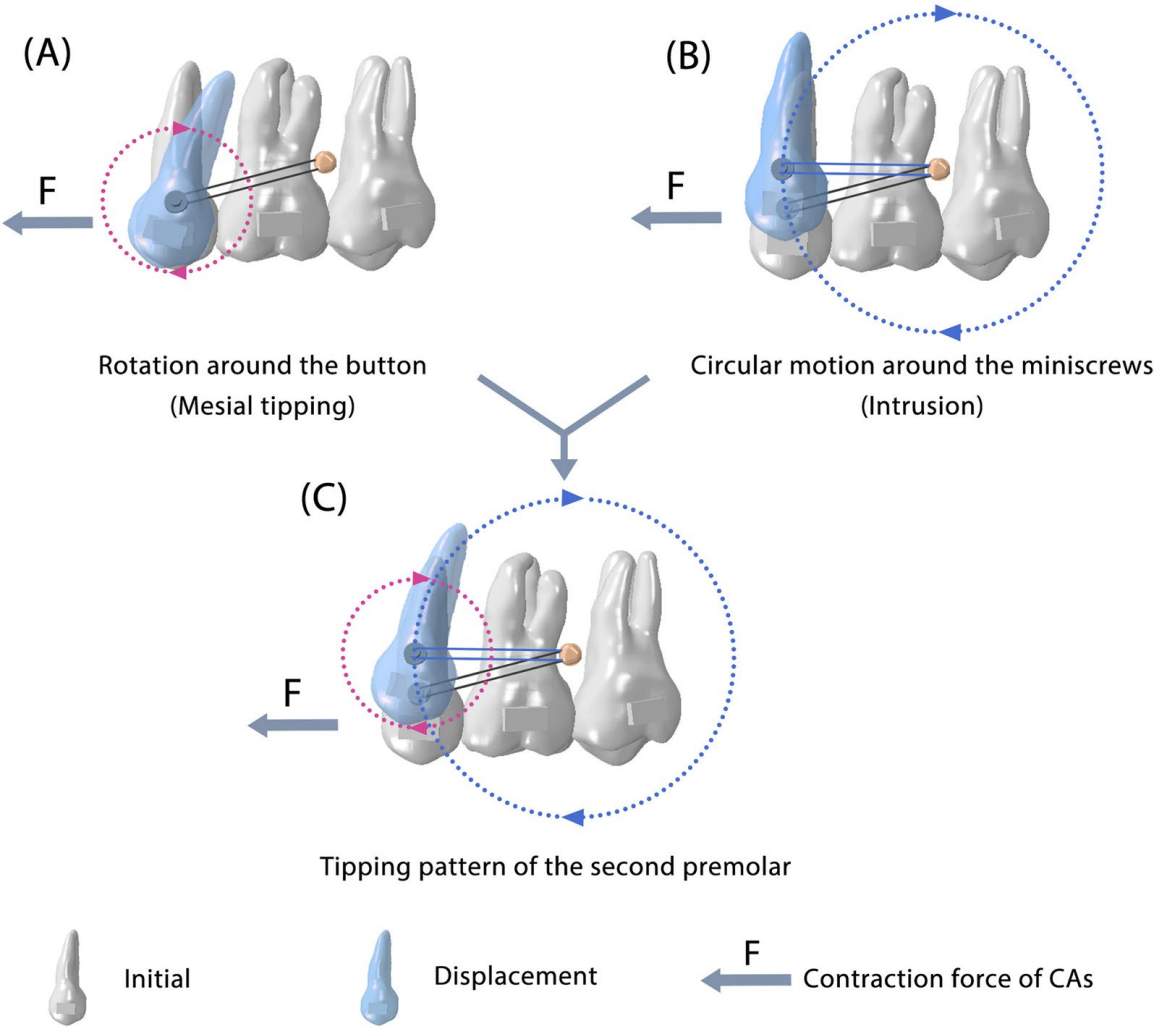
Tipping movement of the second premolars under indirect strong anchorage control can be decomposed into two circular motions. **A** Circular motion around the button. **B** Circular motion around the miniscrew. **C**) Schematic diagram of the integral tipping movement of the second premolars

TADs are indispensable for anchorage management in current extraction cases [22]. Direct and indirect strong anchorages are the two most common TAD-assisted anchorage preservation methods. This study showed that these two anchorages, although only differing in terms of active retraction and passive ligation, did have completely different biomechanical mechanisms. For direct strong anchorage, the elastic force plays an essential role in anchorage control. Typically, in fixed appliance treatments, retraction forces acting on the canine region can cause clockwise rotation of the occlusal plane because the force is located below the center of resistance of the maxillary dentition. Interestingly, when direct anchorage was used in CAs, different mechanical effects were observed, in which the anterior teeth were intruded while the middle of the arch was extruded, causing a counterclockwise rotation of the maxillary occlusal plane and the desired anti-roller-coaster effect (Fig. 4B). This phenomenon is caused by the retraction force acting on the CA, which causes the retraction and intrusion forces on the anterior teeth. Meanwhile, the aligner squeezes in the extraction area, which could further give the posterior teeth a distal tipping force, causing extrusion of the first molar. Finally, an anti-roller-coaster effect can occur (Additional file 1: Fig. S3). Although direct strong anchorage has the desired biomechanical effect, it relies on the compliance of patients. We also found in a previous study that the use of direct anchorage could not prevent anchorage loss in the early stage of wearing CAs because the CA contraction force was much greater than the retraction force. Therefore, the use of direct strong anchorage will have two stages. The first stage is the initial wear of a new CA and elastics, which presents the combined effects of two forces. The overall effect is predominated by contraction force, manifesting as a roller-coaster effect. With the teeth movement, the aligner force decreases, and the daily renewed elastic force becomes prominent, which takes effects on dentition distalization, extruding the middle dental arch and intruding the two ends of the dental arch, presenting the desired effect for resisting the roller-coaster effect. Indirect strong anchorage provided greater posterior tooth anchorage protection, which relies on the distance-limitation effect of metal wire ligation between the second premolar and miniscrew (Fig. 6). Under indirect anchorage control, the second premolars are relatively fixed with miniscrews, which means they can move in a circular motion around the miniscrews. Simultaneously, the second premolars can make a circular motion with the button as the center of rotation. These two circular motions determine the movement of the second premolars and make indirect anchorage a characteristic anchorage control pattern. After understanding the underlying mechanisms of the two anchorage controls, in the following section, we focus on their clinical significance in the bimaxillary space closure process.

For vertical control, because CAs cannot provide sufficient strength for leveling, it is prone to deepen the longitudinal occlusal curve as well as the overbite of the anterior teeth during space closure. In this study, the deep overbite tendency of the anterior teeth was demonstrated by calculating incisor extrusion relative to the posterior teeth (the second premolar and the first molar), defined as “relative incisor extrusion.” The FEA results showed that both of the strong anchorage groups had a deeper longitudinal occlusal curve tendency in the mandibular dentition than in the maxillary dentition. Notably, the mandibular longitudinal occlusal curve was also more easily flattened by the elastic force than the maxillary dentition, which can be attributed to the smaller periodontal ligament area and tooth shape of the lower anterior teeth. Therefore, follow-up visits should focus on controlling the mandibular longitudinal occlusal curve, and proper overcorrection is needed. Indirect strong anchorage was more likely to cause deep overbite of the anterior teeth, which was more suitable for patients with an open bite or shallow overbite. In contrast, the elastic force could relieve incisor extrusion, which is more suitable for patients with a normal or deep overbite.

Sagittal relationships, particularly molar relationships, are critical for orthodontic treatment. As the lower dentition had a greater anterior–posterior teeth anchorage difference than the upper dentition, the lower posterior teeth had less mesial movement than the upper posterior teeth during the same distance of space closure. According to the displacement data (Table 6), the indirect strong anchorage presented the best anchorage protection effect and was the group with the largest bimaxillary anchorage protection difference (approximately twice the difference). Therefore, if indirect strong anchorage control is applied to both the upper and lower dentition, it is likely to have a Class II molar relationship in long-term treatment, which should be considered in the treatment plan and be a focus in follow-up. For direct strong anchorage, the elastic force plays a key role in coordinating the bimaxillary molar relationship. Because the relative anchorage of the upper posterior teeth is relatively weak and the elastic force has a greater anchorage protection effect in the lower posterior teeth (Table 6 and Additional file 1: Table S1), the use of direct strong anchorage could also lead to a Class II molar relationship tendency. To solve this problem, it is recommended to use a greater elastic force or extend the wearing time of the maxillary dentition.

Due to the control deficiency of CAs, tipping movement is the most common movement pattern. However, bodily movements are usually the desired movement patterns in extraction cases. To achieve this, multiple strategies are used to compensate for the control deficiency, such as distal tipping of the posterior teeth before space closure, adding attachments to the teeth, and designing specialized root control attachments [12, 23]. To further facilitate orthodontists in accurately compensating for the control deficiencies of CAs, we analyzed the effect of different anchorages on the rotation center of the tooth. In the indirect strong anchorage group, the button was located at the tooth crown of the second premolars, and the teeth rotated around the button, causing the rotation center in the mesial/distal direction to move toward the tooth crown, which aggravated the mesial tipping of the second premolars (Fig. 6). To address this, it is critical to maintain the position of the second premolar. If the premolars do not detach mesially and remain in a position relative to the molars, the entire posterior teeth will be less mesially displaced. Three key strategies can be used: (1) adding attachments to ensure the retention between the CA and the second premolar; (2) adding force moment to mesially control the root of the posterior teeth; and (3) extruding the premolar and intruding the second molar. We elaborated on these strategies in our previous study. This study further considered the biomechanical differences between bimaxillary dentition. It has been demonstrated that the lower posterior teeth are more prone to losing control, and the overcorrection strategies mentioned above should be strengthened in the lower posterior teeth. For the direct strong anchorage group, the elastic force can add force moment to mesially control the root of the posterior teeth, especially in mandibular posterior teeth. Therefore, the use of direct anchorage is an ideal solution to relieve the tipping movement of posterior teeth; however, it relies on patient compliance.

This study also demonstrated that the two strong anchorage controls had different root control effects on the anterior teeth. The elastic force is beneficial for lingual root control of the anterior teeth, except for the upper central incisors, which may be attributed to the distinct shape of the incisor and upper arch. Therefore, when adopting direct strong anchorage, it is sensible to design more overcorrections to the upper central incisors. The root control effect was enhanced in the lower anterior teeth, which could be attributed to the smaller periodontal ligament area of the lower anterior teeth. However, the lower anterior teeth received the highest stress and experienced the greatest displacement and rotation (Tables 7 and 8). Undoubtedly, risk control and overcorrection of the lower anterior teeth are key in extraction cases. Considering that during space closure, the upper anterior teeth have a larger periodontal ligament area, whereas the lower anterior teeth bear a larger sagittal force, it is difficult to determine which should initially apply for larger torque compensation. Therefore, stage overcorrection is recommended. In the design of the first set of appliances, excessive torque overcorrections can be added to both the upper and lower anterior teeth. During follow-up observations, the amount of overcorrection could be reduced. This approach ensures treatment efficacy while considering the influence of individual patient differences.

**Fig. 7:**
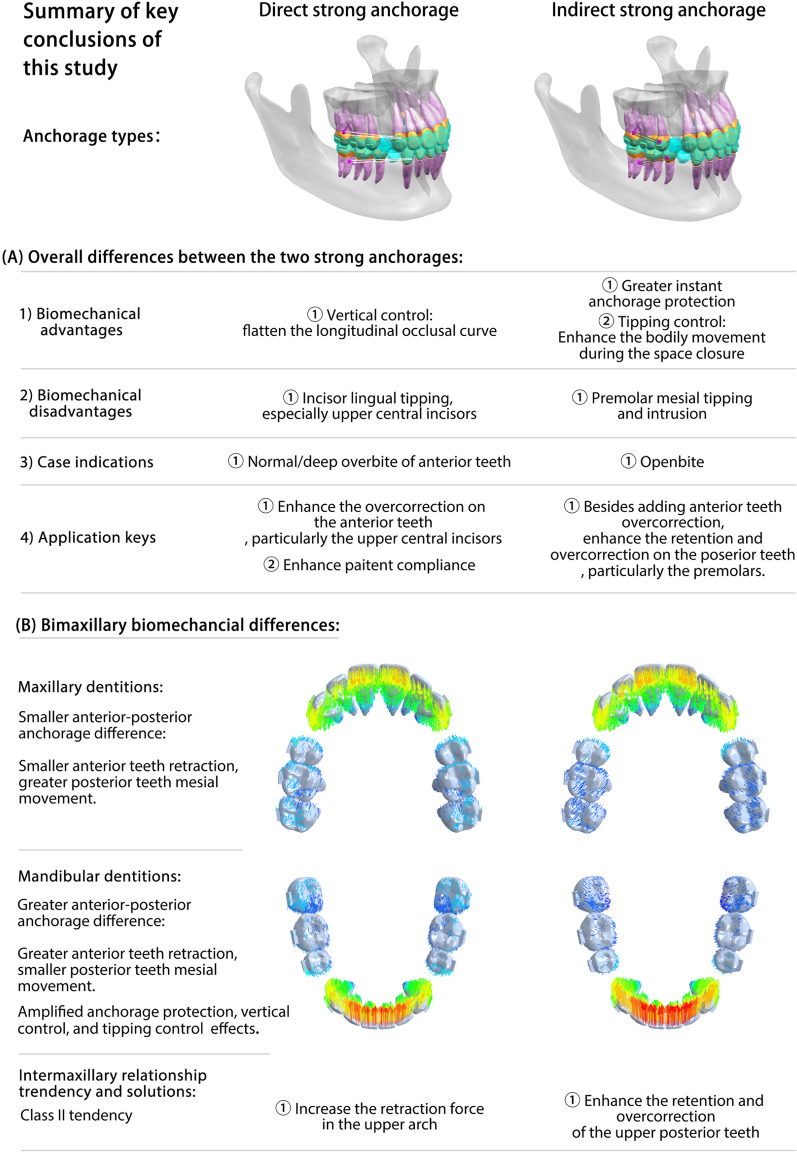
Biomechanical effects of CAs on bimaxillary space closure under two strong anchorages. **A** Overall differences in biomechanical effects between the two strong anchorages. **B** Different biomechanical effects of CAs on bimaxillary space closure under two strong anchorages

The biomechanical effects of different anchorages on the bimaxillary, including their advantages, disadvantages, and solutions, are summarized in Fig. 7.

## Conclusions

Under the three anchorage controls, the bimaxillary dentitions presented different movement patterns during the space closure process by the CAs. Indirect strong anchorage can deepen the longitudinal occlusal curve and is more suitable for patients with an open bite or shallow overbite of the anterior teeth. In contrast, the elastic force is conducive to flattening the longitudinal occlusal curve, which is suitable for patients with a normal or deep overbite of the anterior teeth. Compared with direct strong anchorage, the indirect anchorage presents better anchorage protection and anterior teeth root control effects; however, it requires extra attachments and overcorrection on the posterior teeth, especially the second premolars. Although direct strong anchorage presents better vertical control, compensation strategies, such as overcorrection, are still necessary, especially for the upper central incisors. The upper posterior teeth were more likely to lose anchorage in the two strong anchorage groups. Therefore, enhancing overcorrection and maxillary retraction force should be designed when necessary. The successful use of indirect anchorage is largely dependent on orthodontist design, whereas direct anchorage also relies on patient compliance.

## Supplementary Information

**Additional file 1:** Supplementary figure and table.

## Data Availability

The datasets used and/or analyzed during the current study are available from the corresponding author on reasonable request.

## Abbreviations

CA: Clear aligner
3D: Three-dimensional
TAD: Temporary anchorage device
FEA: Finite element analysis
CBCT: Cone-beam computed tomography
STL: Stereo lithography
