# Supplementary material for: Different biomechanical effects of clear aligners in bimaxillary space closure under two strong anchorages: finite element analysis
Source: Prog Orthod. 2022 Nov 14;23:41. doi: 10.1186/s40510-022-00435-2 (PMC9659682; doi:10.1186/s40510-022-00435-2)
Supplement: Supplementary file 1 — Additional file 1: Supplementary figure and table. [file 40510_2022_435_MOESM1_ESM.doc]

**ADDITIONAL FILE 1**

**Supplementary figure and table**

Different biomechanical effects of clear aligners in bimaxillary space closure under two strong anchorages: Finite element analysis

Jun-qi Liu,^a,b^ Guan-yin Zhu, ^a,b^ Yi-gan Wang,^a,b^ Bo Zhang,^a,b^ Shuang-cheng Wang,^a^ Ke Yao,^a,b^ and Zhi-he Zhao*^a,b^

^a^ State Key Laboratory of Oral Diseases & National Clinical Research Center for Oral Diseases, West China Hospital of Stomatology, Sichuan University, Chengdu, China.

^b^ Department of Orthodontics, West China Hospital of Stomatology, Sichuan University, Chengdu, China.

Jun-qi Liu and Guan-yin Zhu contributed equally to this work.

All authors have completed and submitted the ICMJE Form for Disclosure of Potential Conflicts of Interest, with none reported.

Funding: Research and Development Program, West China Hospital of Stomatology Sichuan University (RD-03-202012), and Sichuan Science and Technology Program (2022ZDZX0031).

Address correspondence to: Zhi-he Zhao, No. 14, 3rd section, RenMinNan Road Chengdu, Sichuan, 610041, P. R. China; e-mail, [zhzhao@scu.edu.cn](mailto:zhzhao@scu.edu.cn)

**

**

**Fig. S1 Thermal contraction method used to stimulate space closure.** A) 1 mm of the clear aligners was selected as the thermal contraction region, which is perpendicular to the arc of the dental arch. B) 0.2 mm of the 1 mm contraction region was completely contracted under the specified thermal shrink condition based on the equation. δ is the linear expansion coefficient of the material (°C); L is the size of the shrink part (mm); Δt is the temperature difference (℃); Δ is the tolerance of the shrink part.

**

**

**Fig. S2 Indicators for analyzing tooth movement.** A) Buccal/lingual or mesial/distal tipping angles were calculated based on the tooth length and displacement value of the crown and root. B) The crown-root movement ratio was used to reflect the position of the center of rotation. The centers of rotation discussed in this study are located between the tooth crown and tooth root apex. Therefore, the larger the ratio, the closer the rotation center is to the root apex, and the greater the root control effect would be. C) The relative incisor extrusion was calculated according to the vertical movement differences between the central incisors and posterior teeth (the second premolar and the first molar), reflecting the change in the longitudinal occlusal curve.


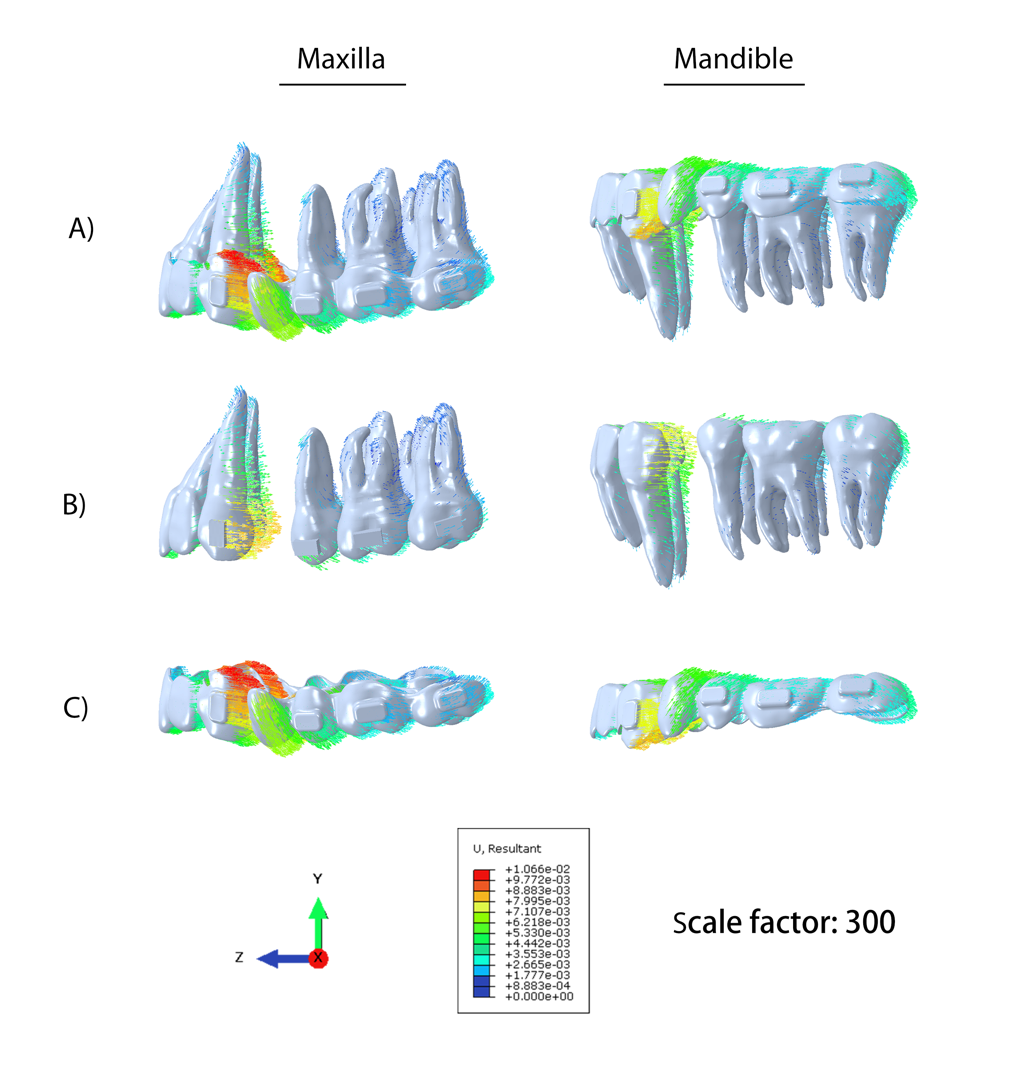


**Fig. S3 Biomechanical effects of 150 g elastic force on clear aligner treatment.** A) Integral biomechanical effects of elastic force; B) Biomechanical effects of 150 g elastic force on teeth; C) Biomechanical effects of 150 g elastic force on clear aligners.

| **Table S1 Teeth movement under mere elastic force (500g)** | | | | | | | | | |
| --- | --- | --- | --- | --- | --- | --- | --- | --- | --- |
| Tooth | Anterior teeth | | | |  | Posterior teeth | | | |
|  | | 1 | 2 | 3 | |  | 5 | 6 | 7 |
| Group | | Maxilla | | | | | | | |
| Extrusion/Intrusion (×10^-2^mm) | | -0.021I | -0.259I | -0.170I | |  | 0.561E | 0.165E | -0.241I |
| Buccal/Lingual movement- crown (×10^-2^mm) | | -0.945L | -0.627L | -1.196L | |  | -0.687L | -0.402L | 0.113B |
| Buccal/Lingual movement- root (×10^-2^mm) | | 0.458B | 0.159B | 0.235B | |  | 0.592B | 0.087B | -0.038L |
| Mesial/Distal movement- crown (×10^-2^mm) | | 0171D | 0.648D | 1.193D | |  | 0.830D | 0.746D | 0.661D |
| Mesial/Distal movement- root (×10^-2^mm) | | -0.079M | -0.379M | -0.362M | |  | -0.570M | -0.394M | -0.342M |
| Relative incisor extrusion to the second premolars (×10^-2^mm) | | -0.582I | | | | | | | |
| Relative incisor extrusion to the first molars (×10^-2^mm) | | -0.186I | | | | | | | |
| Group | | Mandible | | | | | | | |
| Extrusion/Intrusion (×10^-2^mm) | | -0.357I | -0.460I | -0.212I | |  | 0.560E | 0.114E | -0.442I |
| Buccal/Lingual movement- crown (×10^-2^mm) | | -1.044L | -0.945L | -1.354L | |  | -0.401L | -0.202L | -0.007L |
| Buccal/Lingual movement- root (×10^-2^mm) | | 0.136B | 0.049B | 0.270B | |  | 0.229B | 0.089B | 0.003B |
| Mesial/Distal movement- crown (×10^-2^mm) | | 0.124D | 0.599D | 1.192D | |  | 1.008D | 0.915D | 0.799D |
| Mesial/Distal movement- root (×10^-2^mm) | | -0.024M | -0.085M | -0.090M | |  | -0.400M | -0.196M | -0.210M |
| Relative incisor extrusion to the second premolars (×10^-2^mm) | -0.917I | | | | | | | | |
| Relative incisor extrusion to the first molars (×10^-2^mm) | -0.471I | | | | | | | | |
| + values indicate tooth movement in the extrusion, buccal, or distal directions (×10^-2^mm); - values indicate tooth movement in the intrusion, lingual, or mesial directions (×10^-2^mm); E, tooth extrusion based on the occlusal plane; I, tooth intrusion based on the occlusal plane; B, buccal movement; L, lingual movement; M, mesial movement; D, distal movement; crown movement, movement of the body center point of the clinical crown; root movement, root apex point movement; 1, central incisor; 2, lateral incisor; 3, canine; 5, second premolar; 6, first molar; 7, second molar. | | | | | | | | | |
